# Influence of Maternal and Paternal Parenting Style and Behavior Problems on Academic Outcomes in Primary School

**DOI:** 10.3389/fpsyg.2019.00378

**Published:** 2019-03-01

**Authors:** Purificación Checa, Alicia Abundis-Gutierrez, Carolina Pérez-Dueñas, Antonio Fernández-Parra

**Affiliations:** ^1^Department of Development and Education Psychology, Faculty of Educational Sciences, University of Granada, Granada, Spain; ^2^Department of Behavioral Sciences, CUVALLES, University of Guadalajara, Ameca, Mexico; ^3^Department of Psychology, IMIBIC Health Research Institute, Reina Sofía University Hospital of Córdoba, University of Córdoba, Córdoba, Spain; ^4^Department of Personality, Evaluation and Psychological Treatment, Faculty of Psychology, University of Granada, Granada, Spain

**Keywords:** parenting styles, maternal sensitive parenting styles, behavioral problems, academic outcomes, attention, academic achievement

## Abstract

Parents and teachers are concerned about the academic outcomes of children. Among the variables that play an important role in school success, parenting styles and behavior problems are some of the most studied. Literature shows that presence of behavioral problem and parenting styles based on physical punishment, lack of consistency and ineffective limit setting are related to poor academic achievement. The present study examined the influence of maternal and paternal parenting styles and behavior problems on the academic outcomes of primary-school children. Measures used in this study included the Inventory of Parenting Guide, the Child Behavior Checklist and information on academic outcomes (*n* = 78 families). The range age of the students was 6 to 13 years old (mean = 8.08; *SD* = 1.6; 38 girls). The participation rate was 90.7%. The results showed that behavior problems and sensitive parenting style were related to academic outcomes. Specifically, attentional problems and maternal sensitive parenting styles appeared to be significant predictors of academic outcomes in this study. These data suggest the relevance of attention and maternal sensitive parenting styles in understanding processes that promote academic outcomes.

## Introduction

Academic outcome (AO) is an important factor that provides the basis for children’s subsequent life satisfaction. Both parents and researchers are interested in knowing which factors play a crucial role in determining childhood AO. One of the most studied factors is parenting styles (PS). PS is a group of attitudes and behaviors relating to child rearing (Darling and Steinberg, 1993). Bauermeister et al. (1995) defined two PS: (1) sensitive PS (SPS), which is based on warmth, mutual support and appropriate autonomy while maintaining firm and consistent limits on children; and (2) coercive PS, based on physical punishment, lack of consistency and ineffective limit setting. Certain aspects of SPS, such as appropriate autonomy and warmth, facilitate AO and could serve as a secure base from which children can explore their environment and overcome challenges in non-family settings such as at school (Soh-Leong and Lim, 2003; Steinberg et al., 2006; Garn et al., 2010; Masud et al., 2015), while certain characteristics of, such as lack of consistency or ineffective limits, are associated with low AO (Steinberg et al., 1989; Pelegrina et al., 2003; Osorio and Gonzalez-Cámara, 2016; Checa and Abundis-Gutierrez, 2017). Another variable that can affect AO is the presence of behavior problems. Children exhibiting high levels of aggression, anxiety or social problems show poorer AO compared with their low aggressive, low anxiety, sociable peers (Clasen and Brown, 1985; Nelson et al., 1999; Ladd et al., 2006; Schwartz et al., 2006; Checa et al., 2008; Caprara et al., 2014; Carlo et al., 2018). In addition, attentional problems have been found to be both negatively related to AO and a good predictor of AO (Duncan et al., 2007; Breslau et al., 2009). Finally, it is important to note that several studies have shown differences between maternal and paternal reports of their children’s characteristics and abilities (Dumka et al., 2009; Álvarez-García et al., 2016; Llorca et al., 2017).

The current study aims to examine interrelations between PS, behavior problems and AO in primary-school children. Most of studies in the literature have been focused on the parenting practices of mothers. We assessed maternal and paternal PS in order to determine the separate influence of mothers’ and fathers’ PS on AO. We expected to find a positive correlation between maternal and paternal SPS and AO, and either a weak negative or no relation between CPS and AO. Second, we examined how behavior problems were related to AO. We expected behavior problems to be negatively related to AO. Finally, we examined the separate contribution of maternal and paternal reports of behavior problems and parenting styles in predicting AO. Given that mothers are often the primary caregiver, we expected that maternal SPS would be the strongest predictor of AO.

## Methods

### Participants

A total of 78 Spanish families participated in the study. Written informed consent was obtained from the parents of the participants. The mean age of the students was 8.08 years (*SD* = 1.6; 38 girls). The study was carried out in accordance with the Declaration of Helsinki. Ethics approval from University of Granada was obtained.

### Procedure

Four questionnaires, two for the mothers and two for the fathers, were sent by mail together with instructions for completing and returning them to the school. One questionnaire measured behavior problems, the other PS. Finally, teachers provided information on the children’s general AO at the end of the academic year.

### Instruments

#### Inventory of Parenting Guide

*Inventory of Parenting Guide* (Inventario de Prácticas de Crianza) (IPC; Bauermeister et al., 1995). The IPC consists of 37 questions that assess PS in daily situations. Parents’ responses are grouped under two factors: (a) CPS: 15 items that include the use of physical punishment, lack of consistency and ineffective limit-setting; and (b) SPS: 22 items covering warmth, concern, consistency and motivation of children. All responses are recorded on a four-point Likert scale ranging from 0 (never or rarely) to 3 (very frequently).

#### Child Behavior Checklist

*Child Behavior Checklist* (CBCL; Achenbach and Rescorla, 2001). We used the second part of this questionnaire (113 questions) to assess the children’s behavior in everyday situations. Parents’ responses are grouped onto nine scales: anxious/depressed (AD), withdrawn/depressed (WD), somatic complaints (SC), social problems (SP), thought problems (TP), attention problems (AP), rule-breaking behavior (RBB), and aggressive behavior (AB). Responses to all items are recorded on a three-point Likert scale ranging from 0 (never or rarely) to 2 (very frequently).

#### Academic Outcome (AO)

*Academic Outcome* (AO). Teachers provided information on general academic outcomes achieved at the end of the academic year, using the following scores: 0 (failing), 1 (below average), 2 (average), 3 (above average).

#### Missing Data

The valid n for each measure is provided in the Supplementary Material. Sixty-four families consented in writing to providing information about their children’s AO. Twelve fathers refused to complete the CBCL questionnaire, while two mothers and twenty-one fathers did not complete the IPC inventory. Only measures on the CBCL and IPC with an α ≥ 0.60 for fathers and mothers, as well as measures that followed a normal distribution, were included in the analyses (see the Supplementary Material for more information about the material and methods).

## Results

The internal reliability and descriptive analysis are reported in the Supplementary Material.

### Correlations

Pearson’s correlations for behavior problem, PS and AO are presented in Table 1. All behavior problems reported by fathers were negatively related to AO, while only mothers’ reports of WD, AP, and AB were similarly related. Finally, both maternal and paternal SPS were positively related to AO.

**Table 1:** Correlation between measures.

	FATHERS							MOTHERS							
	Behavioral problem					Parenting styles		Behavioral problem						Parenting styles	
	AD	WD	SP	AP	AB	CPS	SPS	AD	WD	SC	SP	AP	AB	CPS	SPS
AO	-0.26^∗^	-0.34^∗^	-0.28^∗^	-0.43^∗∗^	-0.35^∗∗^	0.20	0.36^∗∗^	-0.07	-0.30^∗^	-0.08	-0.05	-0.43^∗∗^	-0.42^∗∗^	0.02	0.33^∗∗^
AD		0.59^∗∗^	0.62^∗∗∗^	0.57^∗∗^	0.64^∗∗∗^	0.01	-0.38^∗∗^		0.57^∗∗^	0.66^∗∗∗^	0.72^∗∗∗^	0.39^∗∗^	0.67^∗∗∗^	-0.26^∗^	-0.04
WD			0.69^∗∗∗^	0.44^∗∗^	0.55^∗∗∗^	-0.02	-0.32^∗^			0.61^∗∗^	0.57^∗∗^	0.29^∗^	0.49^∗∗^	0.19	-0.12
SC											0.56^∗∗^	0.32^∗^	0.53^∗∗^	0.19	-0.09
SP				0.55^∗∗∗^	0.70***	-0.01	-0.38^∗∗^					0.38^∗∗^	0.65^∗∗∗^	0.34^∗∗^	-0.11
AP					0.68^∗∗∗^	-0.05	-0.47^∗∗^						0.54^∗∗^	0.28^∗^	-0.26^∗^
AB						0.01	-0.51^∗∗^							0.34^∗^	-0.26^∗^
CPS							0.12								-0.26^∗^


*AO, academic outcomes; AD, anxious/depressed; WD, withdrawn/depressed; SC, somatic complaints; SP, social problems; AP, attention problems; AB, aggressive behavior; SPS, sensitive parenting styles; CPS, coercive parenting styles. Significance levels: ^∗∗∗^*p* < 0.001; ^∗∗^*p* < 0.01; and ^∗^*p* < 0.05.*

### Regression

We conducted regression analyses to examine the contribution of SPS, and the behavior problems found to be correlated with AO, separately for mothers and fathers. These analyses showed that both maternal and paternal reports of AP, as well as maternal SPS, were significant predictors of AO (Table 2).

**Table 2:** Regression.

	FATHER			Dependent variable	MOTHER		
	Δ*R*^2^ of the model 0.17			Academic outcomes	Δ*R*^2^ of the model 0.15		
	β	t	p		β	T	p
AD	-0.13	-0.78	0.43				
WD	-0.24	-1.5	0.13		0.01	0.1	0.98
SP	0.08	0.51	0.61				
AP	-0.41	-2.9^∗∗^	0.005		-0.27	-2.1^∗^	0.04
AB	-0.17	-0.51	0.61		0.14	0.9	0.34
SPS	0.21	1.3	0.17		0.26	2.1^∗^	0.04


## Discussion

In this study we examined AO in relation to behavior problems and PS. First, we found no negative relation between CPS and AO. This result is consistent with the literature showing either no correlation or a weak one between AO and CPS (Pinquart, 2016; Llorca et al., 2017). As expected, both maternal and paternal SPS were positively related to AO. These results are consistent with those of previous studies (Soh-Leong and Lim, 2003; Steinberg et al., 2006; Garn et al., 2010; Masud et al., 2015). We believe that SPS may serve to provide a secure basis from which children can overcome challenges, for example, in the process of academic learning. Second, as expected we found a negative relation between AO and all behavioral problems reported by the fathers and only some (WD, AP, and AB) reported by the mothers. Previous results have revealed a negative link between AO and behavioral problems in children (Clasen and Brown, 1985; Checa and Rueda, 2011; Llorca et al., 2017; Carlo et al., 2018). Finally, in order to examine the contribution of behavior problems and maternal and paternal PS separately, we ran two separate regression analyses for mothers and fathers. As expected, and as some studies have previously shown (Dumka et al., 2009; Llorca et al., 2017), maternal SPS was a predictor of AO but paternal SPS was not. These results support our prediction and point to the relevance of maternal SPS for AO. It could be hypothesized that fathers and mothers adopt different roles in the education of their children; mothers may have more influence over their children’s activities including academic ones. Future investigations should clarify this question, taking into account paternal and maternal variables such as how much time parents spend with their children or what types of activities they do with them during this time, to determine the real effects of fathers on AO. Finally, one finding that we expected was that AP reported by fathers and mothers was a significant predictor of AO. This data is consistent with previous literature (Duncan et al., 2007; Breslau et al., 2009, 2011). Several studies have shown the benefit of attention on learning school subjects (Bull and Scerif, 2001; Blair and Razza, 2007; Checa et al., 2008). We believe that attention is particularly important for regulating behavior and focusing on what is being discussed in the classroom (Checa and Rueda, 2011). In sum, although the influence of both attention and PS has been investigated previously, this study shows the relevance of both variables as key aspects in supporting children in meeting the demands of school. When children are in the classroom, they must pay attention to their teachers, ignoring distractions in order to concentrate. In this situation, children with high-SPS mothers seem to be confident in their abilities, which could increase their opportunity for effective learning and obtaining a good AO. Also, we believe it is relevant to emphasize the role of AP on AO in order to promote interventions for school-age children that target attention and to work with parents about PS with the aim of improving AO. We believe that social services and a clinical setting could promote family education on SPS practice and more knowledge about behavioral problems related to attention in order to improve AO.

## Limitations and Futures Directions

This study only evaluates the influence of behavioral problems and PS on AO. Future research should study the influence of other variables related to AO, such as interests and intelligence structure (Pellerone et al., 2015, 2017) or interpersonal problems (Lo Coco et al., 2018). Moreover, it would be interesting to study the subjective perception of time (Mannino and Caronia, 2017; Mannino et al., 2017b) and also could be related to AO and to parenting relationship. Future research should replicate these findings using a larger sample and with other types of families (Mannino and Schiera, 2017) or individuals with disabilities (Mannino et al., 2017a) in order to determine whether they are generalizable to the general population. Future studies should also examine familial variables such as socio-economic status and personal variables such as self-regulation, maladaptive personality traits, and well-being (Gervasi et al., 2017; Granieri et al., 2017; Mannino and Faraci, 2017) to clarify the relations found in this study.

## Author Contributions

PC and AF-P designed the work. PC, AA-G, and CP-D analyzed the data. PC and AF-P interpreted the data study. PC, AF-P, AA-G, and CP-D revised the work critically for the important intellectual content and approved the version to be published. All authors affirmed that the questions of the work are appropriately investigated and resolved. PC and AF-P was involved in the conception of the work and the acquisition of the data.

## Conflict of Interest Statement

The authors declare that the research was conducted in the absence of any commercial or financial relationships that could be construed as a potential conflict of interest.
