# Supplementary material for: Influence of Maternal and Paternal Parenting Style and Behavior Problems on Academic Outcomes in Primary School
Source: Front Psychol. 2019 Mar 1;10:378. doi: 10.3389/fpsyg.2019.00378 (PMC6405423; doi:10.3389/fpsyg.2019.00378)
Supplement: Supplementary file 1 [file Data_Sheet_1.PDF]

Supplementary material

Methods

Participants

Five schools were contacted to be part of this study in 2012. One school declined to participate. A total of 86 families agreed to participate. Three families declined to participate, and five families did not meet the inclusion criteria of families with children between 6-13 years old. (see Figure 1). The age range of the students was six to 13 years old (mean= 8,08; SD= 1.6; 38 girls). The participation rate was 90,7%. The participants were from different primary school classes: 16 first-grade students (nine girls), 17 second-grade students (eight girls); 12 third-grade students (three girls); 14 fourth-grade students (five girls), 11 fifth-grade students (seven girls), and eight sixth-grade students (four girls). The age range of the parents was 28 to 52 years old (mean father= 41; SD= 4.5; mean mother= 39; SD= 4.4). With regard to the parents' level of education, 38 had attended elementary school (22 mothers); 32 had attended secondary studies (16 mothers), and 72 had attended university studies (38 mothers). The majority of the students lived with both their father and mother (n= 60), and 18 students lived with their mothers. Most of participants were from Spain (n= 76), and their first language was Spanish. The participants were not paid.

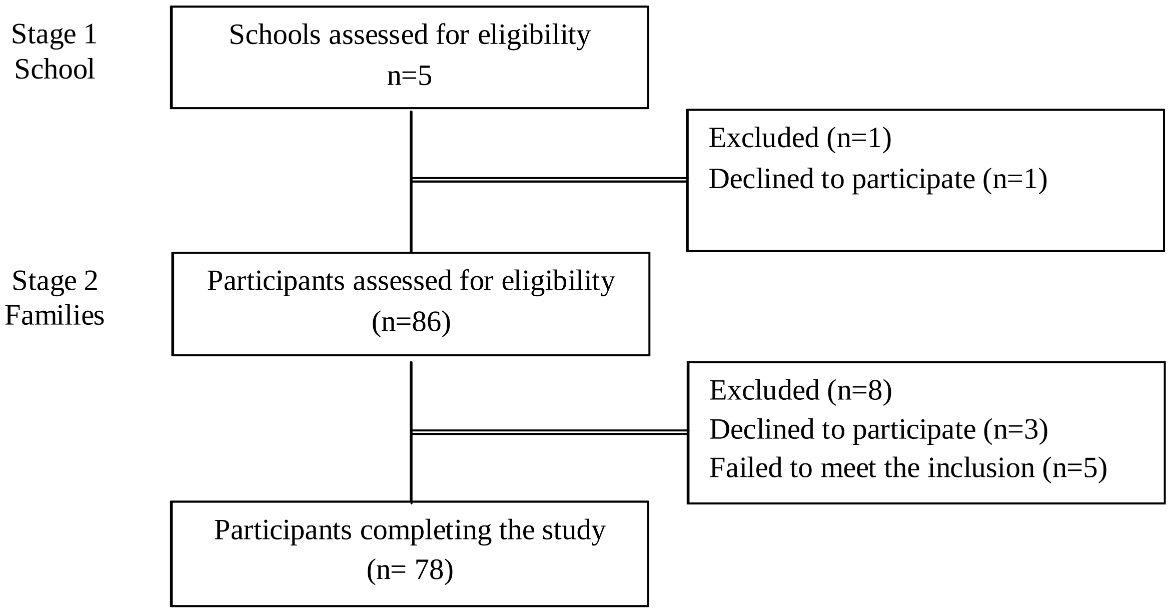

Figure 1. Multistage sampling.

Procedure

Information about the research and the investigator's email address and phone number was sent to the parents together with the four questionnaires. The parents could contact the investigator at any time to clarify any issue related to the research before and after giving written and informed consent.

Instruments

The academic results were evaluated by the teachers following the following criteria ranging from 0 (grades from 0 to 4,9); 1 (grades from 5 to 6,9); 2 (grades from 7 to 8,9) and 3 (grades from 9 to 10).

Results

Descriptive analysis and internal reliability measures.

	n	M (SD)	α	n	SD	α
Academic outcome (AO)				64	2.4 (.60)	
	FATHER			MOTHER		
Anxious/Depressed (AD)	66	.28 (.12)	.63	78	.34 (.12)	.75
Withdrawn (WD)	66	.22 (.13)	.73	78	.26 (.13)	.79
Somatic Complaints (SC)	66	.16 (.12)	.31	78	.22 (.12)	.69
Social Problems (SP)	66	.22 (.12)	.73	78	.24 (.12)	.67
Thought Problems (TP)	66	.10 (.09)	---	78	.15 (.12)	---
Attention Problems (AP)	66	.50 (.15)	.84	78	.53 (.15)	.82
Rule-Breaking Behaviour (RBB)	66	.08 (.07)	---	78	.09 (.09)	---
Aggressive Behaviour (AB)	66	.34 (.16)	.85	78	.35 (.15)	.83
Coercive Parenting Style (CPS)	57	.93 (.15)	.91	76	.60 (.30)	.77
Sensitive Parenting Style (SPS)	57	2.64 (.60)	.67	76	1.69 (.46)	.92

Internal reliability was measured by Cronbach's alpha= α. We did not report the RBB α or TP α here because the samples did not exhibit a normal distribution.
